# Radiocesium contamination and estimated internal exposure doses in edible wild plants in Kawauchi Village following the Fukushima nuclear disaster

**DOI:** 10.1371/journal.pone.0189398

**Published:** 2017-12-14

**Authors:** Rimi Tsuchiya, Yasuyuki Taira, Makiko Orita, Yoshiko Fukushima, Yuukou Endo, Shunichi Yamashita, Noboru Takamura

**Affiliations:** 1 Department of Global Health, Medicine and Welfare, Nagasaki University Graduate School of Biomedical Sciences, Nagasaki, Japan; 2 Department of Nursing, Nagasaki University Graduate School of Biomedical Sciences, Nagasaki, Japan; 3 Kawauchi Municipal Government, Fukushima, Japan; 4 Department of Radiation Medical Science, Atomic Bomb Disease Institute, Nagasaki University Graduate School of Biomedical Sciences, Nagasaki, Japan; University of South Carolina, UNITED STATES

## Abstract

Kawauchi Village, in Fukushima Prefecture, is located within a 30-km radius of the nuclear disaster site of the Fukushima Daiichi Nuclear Power Plant (FDNPP). “*Sansai”* (edible wild plants) in this village have been evaluated by gamma spectrometry after the residents had returned to their homes, to determine the residents’ risk of internal exposure to artificial radionuclides due to consumption of these plants. The concentrations of radiocesium (cesium-134 and cesium-137) were measured in all 364 samples collected in spring 2015. Overall, 34 (9.3%) samples exceeded the regulatory limit of 100 Bq/kg established by Japanese guidelines, 80 (22.0%) samples registered between 100 Bq/kg and 20 Bq/kg, and 250 (68.7%) registered below 20 Bq/kg (the detection limit). The internal effective doses from edible wild plants were sufficiently low (less than 1 mSv/y), at 3.5±1.2 μSv/y for males and 3.2±0.9 μSv/y for females (2.7±1.5 μSv/y for children and 3.7±0.7 μSv/y for adults in 2015). Thus, the potential internal exposure doses due to consumption of these edible wild plants were below the applicable radiological standard limits for foods. However, high radiocesium levels were confirmed in specific species, such as *Eleutherococcus sciadophylloides* (“*Koshiabura”*) and *Osmunda japonica* (Asian royal fern, “*Zenmai”*). Consequently, a need still might exist for long-term follow-up such as environmental monitoring, physical and mental support to avoid unnecessary radiation exposure and to remove anxiety about adverse health effects due to radiation. The customs of residents, especially the “*satoyama*” (countryside) culture of ingesting “*sansai*,” also require consideration in the further reconstruction of areas such as Kawauchi Village that were affected by the nuclear disaster.

## Introduction

On March 11, 2011, the 9.0-magnitude Great East Japan Earthquake and subsequent tsunami caused extensive damage to the Fukushima Daiichi Nuclear Power Plant (FDNPP), operated by the Tokyo Electric Power Company (TEPCO). The result was a severe nuclear accident and radioactivity release to the atmosphere from the FDNPP [1]. The released radionuclides were deposited on land and on the ocean around the FDNPP. The estimated total amount of iodine-131 (^131^I) released ranged from about 100 to about 500 petabecquerel (PBq) and that of cesium-137 (^137^Cs) was generally in the range of 6–20 PBq [2]. These ranges comprised about 2–8% of the total inventory of ^131^I and about 1–3% of the total inventory of ^137^Cs in the three operating reactors (Units 1–3) at the time of the accident. For perspective, the estimated releases of ^131^I and ^137^Cs from FDNPP were about 10% and 20%, respectively, of the releases estimated for the Chernobyl accident [2]. These two radionuclides, together with cesium-134 (^134^Cs), made by far the largest contribution to the exposure of the public [2]. The total release of radionuclides to the atmosphere estimated by the United Nations Scientific Committee on the Effects of Atomic Radiation (UNSCEAR) for ^131^I, ^134^Cs, and ^137^Cs were 120.0, 9.0, and 8.8 PBq, respectively [2].

During the six years since the FDNPP accident, the levels of environmental radioactivity have been decreasing due to the natural decay of the radionuclides, to erosion and to ecosystem effects [3,4]. The Japanese Government and local governments have also made efforts to develop strategies and plans for implementing remediation activities, to restore the living conditions of the residents affected by the nuclear accident, and to enable the return of evacuated people [5].

Kawauchi Village is located within a 30-km radius from FDNPP. On January 31, 2012, the village mayor declared that residents who lived at least a 20-km radius away from the FDNPP could return to their homes because the Japanese Prime Minister had declared that the reactors had achieved a state of cold shutdown in December 2011, and the radiation doses were at comparatively low levels [6,7]. Five years have passed since this “declaration of return,” and 2,185 (80.4%) of the 2,717 registered residents have returned to the village as of May 1, 2017 [8]. However, only a limited number of the evacuated residents who had lived in areas closer to the FDNPP, where evacuation orders were lifted between 2012 and 2017, have returned, due to anxieties about potential health effects caused by exposure to radiation derived from the accident [9,10]. In addition to the risk of external exposure to the whole body, the risk of internal exposure during the residents’ daily activities remains a particular matter of public attention, thereby necessitating some means to inform the public regarding safety [9–17].

**Fig 1:**
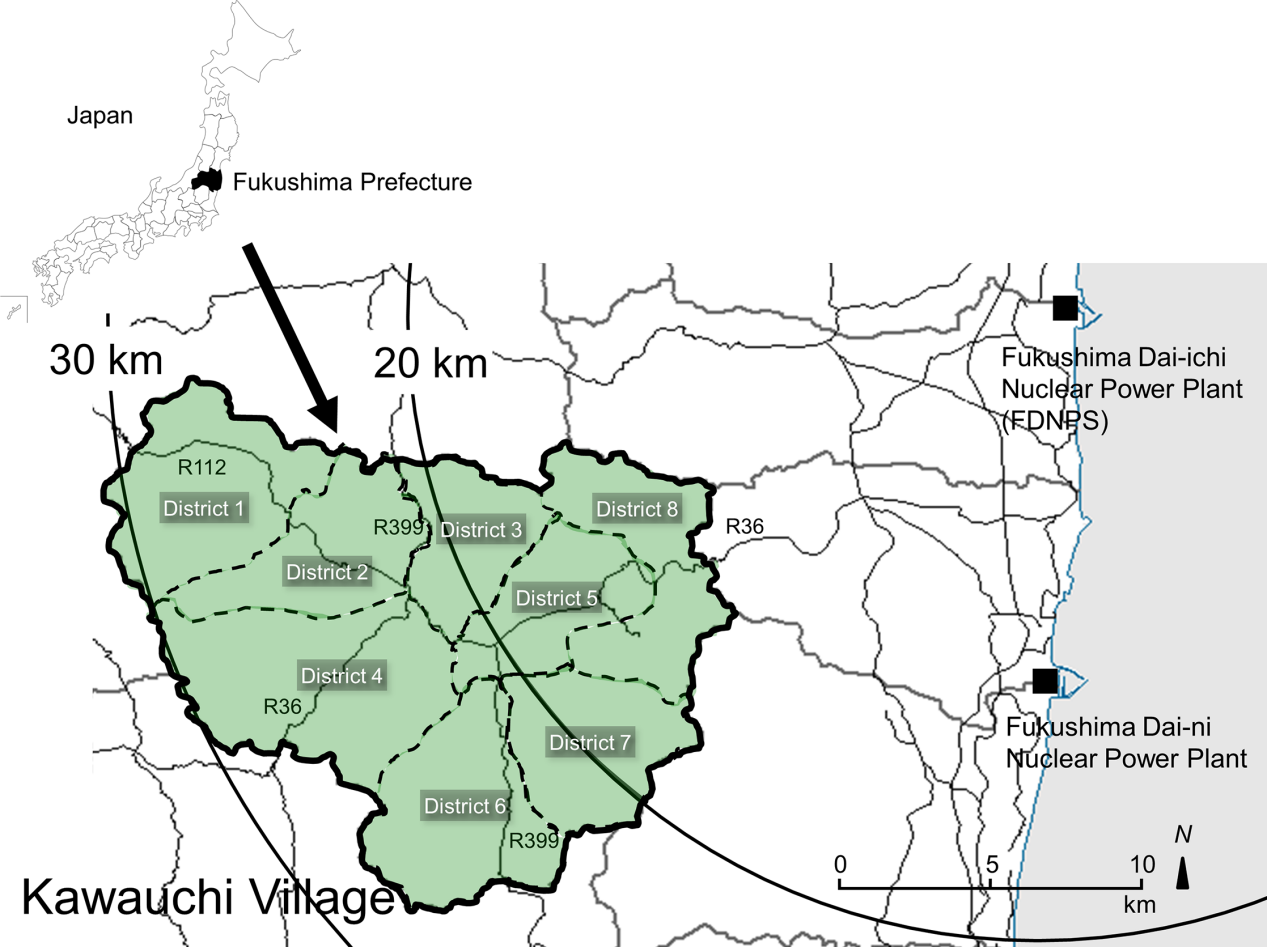
Location of Kawauchi Village, Fukushima Prefecture, Japan. Kawauchi Village is located within a 30-km radius of the FDNPP. The second author (Y.T.) created the map using the software (Green Map Ⅲ^®^, Tokyo Shoseki CO., LTD, Tokyo, Japan. http://www.tokyo-shoseki.co.jp/company_english/philosophy.html). Reprinted from GreenMap Ⅲ^®^ under a CC BY license, with permission from Tokyo Shoseki Co., Ltd.; original copyright 2003.

In Japan, *sansai* (edible wild plants) are traditionally consumed in a variety of ways as *satoyama* (countryside) culture [18–20]. Many plant parts (roots, leaves, stems, buds, seeds, flowers, etc.) of edible wild plants are very nutritious, and these traits are pronounced in plants growing in regions of Eastern Japan that receive snow in winter, including Fukushima Prefecture [18–20]. However, few reports have documented the internal radiation exposure to humans who consume seasonal and edible wild plants. Although our previous reports showed that residents who returned to Kawauchi Village experienced only limited internal exposure, we also confirmed that radiocesium in wild mushrooms collected at Kawauchi Village from 2014 through 2015 exists in several species at high radioactivity levels (Bq/kg) [21–25]. In the current research, we used gamma spectrometry to analyze the concentrations of artificial radionuclides such as radiocesium in edible wild plants collected in the forests of Kawauchi Village in order to evaluate the internal exposure risk derived from the FDNPP accident (**Fig 1**).

## Materials and methods

### Sampling sites

The Fukushima Daiichi Nuclear Power Plant is located on the east coast of Honshu Island, approximately 200 km northeast of Tokyo, Japan. Samples were collected at Kawauchi Village (the public office, N37° 20', E140° 48'), 22.0 km southwest of the FDNPP (N37° 25', E141° 02') (**Fig 1**).

Personnel at the Kawauchi Village Office have been conducting a radioactive contaminant survey for local foods since their return to their homes. In the current research, 364 edible wild plants samples (approximately 200–2,000 g fresh weight) collected at Kawauchi Village during April to June in 2015 were placed in polyethylene bags.

### Measurement of radionuclides

All measurements were performed at Kawauchi Village, Fukushima Prefecture, Japan. The activity concentrations of artificial radionuclides (^131^I, ^134^Cs and ^137^Cs) were analyzed in all 364 samples of edible wild plants (**Tables 1** and **2)**. All collected samples were cleaned of contaminants, such as soil, gravel, and tree stems and branches, washed with water, and stored refrigerated for several days. After crushing with a mixer, the samples were placed in acrylic acid resin containers, weighed (approximately 200–500 g fresh weight), measured for density and height, and analyzed for 1,800 s (real time) using a ϕ2×2 inch thallium-doped sodium iodide (NaI) detector (Canberra^®^, CAN-OSP-NAI, AREVA NC Inc., Meriden, CT, USA) coupled to a multi-channel analyzer (Genie 2000, Canberra Japan KK., Tokyo, Japan) covering 1,024 channels (2,000 keV). We set the measuring time to detect objective radionuclides such as radiocesium. The gamma-ray peaks used for measurements were 604.7 keV for ^134^Cs (2.1y) and 661.6 keV for ^137^Cs (30y). The resolution of the instrument was < 7.5% on 661.64 keV for ^137^Cs. Furthermore, the sum peak of ^134^Cs (1,401 keV) was separated from the gamma-ray peak of potassium-40 which is often dominant in natural radionuclides. We adopted the LabSOCS (Laboratory Sourceless Calibration Software by Canberra^®^) mathematical efficiency calibration software for geometric and detector efficiency, which calculates the calibration without the need for the standard activity calibration sources normally used for efficiency calibration. Sample collection, processing, and analysis were executed in accordance with the standard methods of radioactivity measurement recommended by the Ministry of Education, Culture, Sports, Science, and Technology in Japan [26,27].

**Table 1:** Summary of the radiocesium survey of edible wild plants collected at Kawauchi Village, Fukushima prefecture, during April–June, 2015.

District	*n*	<20 Bq/kg fresh weight^a^	20–100 Bq/kg	>100 Bq/kg
1	74	58	13	3
2	33	24	8	1
3	19	15	4	0
4	33	26	3	4
5	96	61	26	9
6	45	34	4	7
7	19	12	5	2
8	45	20	17	8
Total	364 (100)^b^	250 (68.7)	80 (22.0)	34 (9.3)

^a^Not detected (detection limit for ^134+137^Cs: <20 Bq/kg-fresh).

^b^Percentage

**Table 2:** Summary of the radiocesium survey of edible wild plants collected at Kawauchi Village, Fukushima prefecture, duringApril–June, 2015.

Species (*in Japanese*)	*n*	<20 Bq/kg fresh weight^a^	20–100 Bq/kg	>100 Bq/kg
*Petasites japonicus* (*“Fuki”*)	85	77 (90.6)^b^	8 (9.4)	0 (0)
*Aralia cordata* (*“Udo”*)	92	76 (82.6)	15 (16.3)	1 (1.1)
*Pteridium aquilinum* (*“Warabi”*)	84	44 (52.4)	30 (35.7)	10 (11.9)
*Phyllostachys edulis* (*“Takenoko”*)	35	21 (60.0)	12 (34.3)	2 (5.7)
*Aralia elata* (*“Taranome”*)	10	3 (30.0)	1 (10.0)	6 (60.0)
*Parasenecio delphiniifolius* (*“Shidoke”*)	17	9 (52.9)	7 (41.2)	1 (5.9)
*Matteuccia struthiopteris* (*“Kusasotetsu”*: *“Kogomi”*)	13	9 (69.2)	3 (23.1)	1 (7.7)
*Artemisia indica* var. *maximowiczii* (*“Yomogi”*)	3	3 (100)	0 (0)	0 (0)
*Eleutherococcus sciadophylloides* (*“Koshiabura”*)	7	0 (0)	0 (0)	7 (100)
*Osmunda japonica* (*“Zenmai”*)	10	2 (20.0)	2 (20.0)	6 (60.0)
*Hosta sieboldiana* (*“Urui”*)	3	3 (100)	0 (0)	0 (0)
*Fallopia japonica* (*“Itadori”*)	5	3 (60.0)	2 (40.0)	0 (0)
Total	364 (100)	250 (68.7)	80 (22.0)	34 (9.3)

^a^Number of not detected samples (detection limit for ^134+137^Cs: <20 Bq/kg-fresh).

^b^Percentage.

### Effective dose

**Fig 2:**
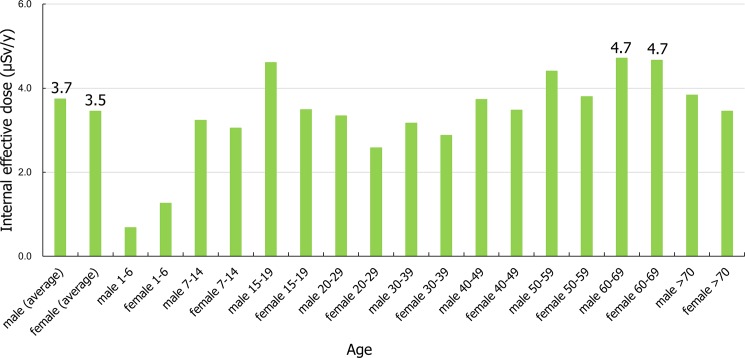
Internal effective doses due to radiocesium in Kawauchi Village, Fukushima prefecture in 2015. The internal effective doses from edible wild plants were 3.5±1.2 μSv/y for males and 3.2±0.9 μSv/y for females (2.7±1.5 μSv/y for children and 3.7±0.7 μSv/y for adults).

After the measurements, the internal effective doses (estimated effective doses) from the edible wild plants samples were estimated from the radioactive fission product concentrations, using the following formula:

Hint=C∙Dint∙e (1)

where *C* is the activity concentration (the median value of 12 varieties of edible wild plants) of the detected artificial radionuclides (i.e., radiocesium) (Bq/kg fresh weight) (**Table 3**). Here, *Dint* is the dose conversion coefficient for child intake (age: 0–18 years, 1.3×10^−5^ to 2.6×10^−5^ mSv/Bq for ^134^Cs and 9.6×10^−6^ to 2.1×10^−5^ mSv/Bq for ^137^Cs) and for adult intake (age: > 19 years, 1.9×10^−5^ mSv/Bq for ^134^Cs and 1.3×10^−5^ mSv/Bq for ^137^Cs), as per the International Commission on Radiological Protection (ICRP) 1996 [28], and *e* is quoted from the median value of daily intake data (g/man/day) for age and gender, as issued by the Ministry of Health, Labour, and Welfare (MHLW), Japan in 2015 [29]. The estimated internal effective doses were calculated by assuming that the undetected data of the radiocesium had activity concentrations corresponding to one-half of the detection limit for edible wild plant samples in 2015 (the rates of undetected data were in a range of 60–80%) (**Tables 1** and **2**). These assumptions were based on the Global Environment Monitoring System-Food Contamination Monitoring and Assessment Programme (GEMS/Food) by the World Health Organization (WHO) and the new standard limits for radionuclides in food recommended by the MHLW [30–32]. In the current research, edible wild plants were selected to calculate the estimated effective doses because these samples were routinely collected and are assumed to contribute to the potential for internal exposure. Therefore, the estimated effective internal doses were estimated by assuming that the residents usually ingested edible wild plants during the investigation period (3 months or a year) after they returned to their homes in Kawauchi Village (**Table 4** and **Fig 2**).

**Table 3:** Distribution of radiocesium in edible wild plants in Kawauchi Village, Fukushima prefecture, during April–June, 2015.

Species (*in Japanese*)	*n*	Range (Bq/kg fresh weight)		Mean (Bq/kg)		Median (Bq/kg)	
		^134^Cs	^137^Cs	^134^Cs	^137^Cs	^134^Cs	^137^Cs
*Petasites japonicus* (*“Fuki”*)	85	<18–33	<10–27	11±2.9^a^	11±2.8	10 (10)^b^	10 (10)
*Aralia cordata* (*“Udo”*)	92	<10–14	<11–46	11±3.9	14±19	10 (10)	10 (17)
*Pteridium aquilinum* (*“Warabi”*)	84	<10–513	<11–647	23±60	38±84	10 (30)	10 (82)
*Phyllostachys edulis* (*“Takenoko”*)	35	<18–34	<10–28	14±18	32±68	10 (10)	10 (45)
*Aralia elata* (*“Taranome”*)	10	<21–201	<18–771	60±68	216±266	28 (178)	103 (673)
*Parasenecio delphiniifolius* (*“Shidoke”*)	17	<10–33	<10–96	13±5.8	26±25	10 (18)	10 (61)
*Matteuccia struthiopteris* (*“Kusasotetsu”*: *“Kogomi”*)	13	<12–22	<12–87	11±3.2	20±22	10 (12)	10 (44)
*Artemisia indica* var. *maximowiczii* (*“Yomogi”*)	3	n.d.^c^	n.d.				
*Eleutherococcus sciadophylloides* (*“Koshiabura”*)	7	30–2,084	140–7,374	624±633	2,188±2,246	512 (1,219)	1,904 (4,277)
*Osmunda japonica* (*“Zenmai”*)	10	<23–155	<12–643	40±42	146±182	28 (67)	101 (274)
*Hosta sieboldiana* (*“Urui”*)	3	n.d.	n.d.				
*Fallopia japonica* (*“Itadori”*)	5	n.d.	<13–23		13±5.0		10 (19)
Total	364	<10–2,084	<10–7,374	28±126	72±438	10 (22)	10 (57)

^a^Data are presented as mean±SD. Error shows one sigma standard deviation from counting statistics.

^b^90th percentile.

^c^Not detected (detection limit for ^134+137^Cs: <20 Bq/kg-fresh).

**Table 4:** Estimated internal effective doses from edible wild plants due to radiocesium in Kawauchi Village, Fukushima prefecture.

Period	Estimated dose	Gender	Mean	Average	Age Group^a^								
					1–6	7–14	15–19	20–29	30–39	40–49	50–59	60–69	> 70
April–June, 2015	μSv/3 month	Male	0.9±0.3^b^	0.9	0.2	0.8	1.2	0.8	0.8	0.9	1.1	1.2	1.0
		Female	0.8±0.2	0.9	0.3	0.8	0.9	0.6	0.7	0.9	1.0	1.2	0.9
	μSv/y^c^	Male	3.5±1.2	3.7	0.7	3.2	4.6	3.3	3.2	3.7	4.4	4.7	3.8
		Female	3.2±0.9	3.5	1.3	3.0	3.5	2.6	2.9	3.5	3.8	4.7	3.5

^a^Dose conversion coefficients are from Internal Dose Easy Calculation Code by ICRP Publ.72.

^b^Data are presented as mean±SD.

^c^Values corresponded to the annual internal exposure dose.

## Results

A summary of the radioactive contaminants survey for edible wild plants collected at Kawauchi Village is shown in **Table 1** and **Table 2**. Thirty-four (9.3%) samples exceeded 100 Bq/kg, 80 (22.0%) samples registered between 100 Bq/kg and 20 Bq/kg, and 250 (68.7%) samples registered below 20 Bq/kg (the detection limit). The distribution of radiocesium concentrations in edible wild plants in Kawauchi Village is shown in **Table 3**. Although radiocesium was not detected in many samples, the radiocesium for some species, such as *Eleutherococcus sciadophylloides* (“*Koshiabura”*) and *Osmunda japonica* (“*Zenmai”*), were often detected in high concentrations in every year. The radiocesium concentrations of *E*. *sciadophylloides* were 624±633 Bq/kg for ^134^Cs (ranging from 30 to 2,084 Bq/kg) and 2,188±2,246 Bq/kg for ^137^Cs (ranging from 140 to 7,374 Bq/kg).

The estimated internal effective doses from edible wild plants due to radiocesium in Kawauchi Village are summarized in **Table 4** and **Fig 2**. These values were 3.5±1.2 μSv/y for males and 3.2±0.9 μSv/y for females (2.7±1.5 μSv/y for children and 3.7±0.7 μSv/y for adults) during the investigation period.

## Discussion

Although comparatively higher concentrations of radiocesium were observed in some samples in the current research, the detection rate of radiocesium levels greater than 100Bq/kg (the radiocesium regulatory limit for general foods in Japan) was 9.3%. Our current results suggest that radiocesium concentrations remained at extremely low levels in 2015. By contrast, some edible wild plants such as *Aralia elata* (“*taranome”*), *E*. *sciadophylloides*, and *O*. *japonica* showed median concentrations of radiocesium (^134+137^Cs) that exceeded the regulatory limit. Especially, the radiocesium concentrations of *E*. *sciadophylloides* were much higher than the regulatory limit (^134^Cs: 512 Bq/kg-fresh weight; ^137^Cs: 1,904 Bq/kg-fresh weight by median). The Ministry of Agriculture, Forestry and Fisheries of Japan prohibits the collection of *E*. *sciadophylloides* in places where the air dose is greater than 0.2 μSv/h because high concentrations of radiocesium have been often found in *E*. *sciadophylloides* around the FDNPP since the spring of 2013 [11,19,33]. Thus, the current research suggests that radiocesium concentrations in *E*. *sciadophylloides*, including plants growing in Kawauchi Village, remain higher than in other edible wild plants around the FDNPP.

The current research also provided estimates of the estimated internal effective doses from edible wild plants in Kawauchi Village. The internal exposure risk by intake of agricultural products such as vegetables, edible wild plants and wild mushrooms is a matter of concern for residents. Edible wild plants including wild mushrooms are commonly ingested because approximately 90% of the area of Kawauchi Village is covered by forests andfarming is the dominant industry in the region. Thus, the daily consumption (g/day) of edible wild plants would likely contribute to the residents’ estimated internal effective doses from other sources (see Eq (1)). The current research suggests that dietary habits between generations (i.e., old versus young age) contributed to the estimated internal effective doses [25,29]. In contrast, gender did not always contribute to these doses except the group aged 1 to 6 years [25,29]. Persons fifty years of age and older and the group aged 15 to 19 years tended to have slightly higher consumption of edible wild plants when compared to the other age groups, so that the estimated internal effective dose was extremely low in the group aged 1 to 14 years and in the group 20 to 39 years [29].

The results from the current research also suggested that the cumulative internal exposure risk experienced by ingesting edible wild plants was very low, although the potential for radiocesium exposure still exists [21,24,25,34,35]. The internal effective doses ranged from 0.12–11.94 μSv for males and from 0.17–10.15 μSv for females (from 0.04–4.57 μSv for male children and from 0.50–7.77 μSv for female children) from April 2013 to December 2014, and ranged from 2.3–9.8 μSv/y for males and from 2.9–12 μSv/y for females (from 2.3–12 μSv/y for male children and from 2.9–12 μSv/y for female children) from May 2012 to March 2013 due to the ingestion of edible wild plants and mushrooms [24,25]. This tendency shown for the annual effective doses from radiocesium in foods is consistent with the estimations made by the Nuclear Regulation Authority (NRA) and other estimates reported elsewhere [6,7,11]. In our research, we evaluated the internal exposure doses and the long-term variation based on radiocesium concentrations of edible wild plants, and we found no obvious variation in radiocesium contamination between 2012 and 2015 [24,25]. The cumulative internal effective doses from edible wild plants due to radiocesium were 21.1±4.00 μSv/4y for males and 20.2±3.58 μSv/4y for females (16.0±7.18 μSv/4y for children and 23.0±3.74 μSv/4y for adults) between 2012 and 2015 (Table A in S1 Appendix) [24,25]. Additionally, significant differences were observed in the estimated internal effective doses between males in 2013 and females in 2015, females in 2013 and females in 2015, and females in 2014 and females in 2015 (p<0.001) during the investigation period (Fig A in S1 Appendix) [24,25]. Thus, current internal doses are estimated to be sufficiently low when compared to the public dose limit (1 mSv/y, ICRP, 1991). Moreover, the monitoring system for local foods appears to be functioning effectively as a risk evaluation tool in Kawauchi Village. However, the finding of extremely high radiocesium concentrations in several species such as *E*. *sciadophylloides* suggests that precautions should be taken when ingesting these wild plants (**Table 3**) [11,19]. This species-level analysis of edible wild plants is likely of scientific and public health importance for the long-term assessment of environmental impacts in Fukushima.

Conversely, external exposure doses in areas located northwest of the FDNPP, including ‘difficult-to-return zones’ such as Iitate Village over 30 km from FDNPP, are now at low levels (less than 0.5–19.0 μSv/h in 2015; equivalent to approximately 2.5–95 mSv/y) [6,7]. The data collected by national institutions such as the NRA and in our previous research have confirmed that the areas located northwest of FDNPP, including Iitate Village, were clearly affected by the FDNPP accident when compared with other regions in Fukushima prefecture [5–7,36]. Multiple artificial radionuclides, including radiocesium, were transported by adhesion to aerosol or soil particles because weather conditions, such as rain, snow, and wind, likely facilitated the transport of radionuclides immediately after the FDNPP accident [5–7,36]. Nevertheless, the external exposure doses are now at low levels in most areas of Kawauchi Village (less than 0.2–0.5 μSv/h in 2015; equivalent to approximately 1–2.5 mSv/y), according to airborne monitoring data by the NRA [7]. External effective doses due to artificial radionuclides derived from the FDNPP accident in Kawauchi Village were extremely low compared to the heavily contaminated areas located northwest of the FDNPP [7,33]. Current estimates also indicate that the external exposure doses in Kawauchi Village due to artificial radionuclides derived from the FDNPP accident were equivalent to the public dose limit and/or were sufficiently lower than the existing exposure situation (20 mSv/y) determined by the ICRP. Moreover, the monitoring system for local foods works effectively in the areas around the FDNPP and the internal exposure risk may be strictly controlled, although a significant external exposure risk still exists in areas where evacuation orders have been lifted [32].

Forests (approximately 68%) cover much of Fukushima Prefecture [37]. Most of the radiocesium was initially intercepted by the tree canopies during the early stage of contamination from the FDNPP accident and much of the radiocesium has been transported to the ground surface during the intervening time [38–40]. The radiocesium found in plants may also have been leached by the rain and accumulated on the surface of the soil. The importance of the processes for removal of radiocesium from the tree canopies decreased in the order of litterfall, throughfall, and stemflow [38–40]. The radiocesium activity fraction was also observed in old foliage within the tree compartments [38–40]. On the other hand, edible wild plants with roots that develop in shallow soil may take up radiocesium by these roots when the radiocesium enters the forest ecosystem [19]. The mechanisms responsible for the increase in radiocesium concentrations in edible wild plants is not clear [19]. Further investigation of the Fukushima forests are necessary to evaluate variation of ecosystem cesium transport processes under various soil-landscape conditions [38–40]. The internal exposure doses due to consumption of edible wild plants might decrease slower than the external exposure doses and they might also show irregular variation with time, although the biological and effective half-lives of radiocesium are less than 100 days because they are distributed in whole body muscles [35,41,42]. Conversely, consumption of edible wild plants, such as *E*. *sciadophylloides*, should be minimized to reduce internal exposure to radiocesium from the viewpoint of risk management. A long-term follow-up study of dietary exposure is required to evaluate annual dietary consumption of radiocesium in areas around the FDNPP.

The current research has several limitations. One limitation is that the sample size (*n* = 364) might not be large enough because the radioactivity in edible wild plants will vary widely with soil properties and/or the amount of radiocesium accumulated immediately after the FDNPP accident. However, the concentration of radiocesium was measured in 1,700 samples collected in Kawauchi Village from 2012 through 2015 and these data are consistent with the values reported here (Table B and C in S1 Appendix)[24,25]. Moreover, the intake of radiocesium varies by cooking method and consumption habits and edible wild plants are eaten a variety of ways (boiled, fried and so on). Traditionally, edible wild plants are also eaten raw in Japan. Thus, it is necessary to calculate effective doses under a variety of conditions. In the current research, internal exposure risks were evaluated using raw edible wild plants which the inhabitants in Kawauchi Village consume daily. Unfortunately, we were not able to investigate the precise distribution of *E*. *sciadophylloides* that showed high radiocesium concentrations because of an insufficient number of *E*. *sciadophylloides* samples collected during the investigation period. Also, some radionuclides could not be analyzed using our procedure, including strontium-90 (^90^Sr) (29 y) and plutonium (Pu). However, both Sr and Pu show limited distributions around the FDNPP according to the data of national institutions such as the NRA [7].

In conclusion, we evaluated the radiocesium concentrations in edible wild plants to estimate internal exposure doses around the FDNPP. Our findings suggest that the internal exposure doses depend on the dietary habits of consumers and the species of edible wild plants consumed, with some age groups getting larger doses than others. However, based on our observations, it is predicted that overall the internal doses were significantly lower than recommended the public dose limit (1 mSv/y) (**Tables 3** and **4**). Radiocesium concentrations were relatively high in some edible wild plant species, such as *E*. *sciadophylloides*; therefore, consumption of this plant as well as mushrooms should be avoided [11,21,29]. Nevertheless, the monitoring system for local foods is effective as a risk communication tool in Kawauchi Village. The FDNPP nuclear disaster has necessitated long-term follow-up of environmental monitoring and countermeasures like further decontamination and restriction of the ingestion of foods that can cause unnecessary radiation exposure, and physical and mental health support services [43–45]. Sustained diligence is needed to protect the physical and mental health of Fukushima residents while allowing for their “*satoyama*” (countryside) culture of ingesting "*sansai*" (edible wild plants) during the remediation of radiation-affected areas, such as Kawauchi Village, following the nuclear disaster.

## Supporting information

S1 Appendix Estimated internal effective doses from edible wild plants due to radiocesium in Kawauchi Village, Fukushima prefecture, during 2012–2015 (Table A).
Variations of estimated internal effective doses from edible wild plants due to radiocesium in Kawauchi Village, Fukushima Prefecture, during 2012–2015 (Fig A).
Summary of the radiocesium survey of edible wild plants (12 species) during 2012–2015 (Table B).
Distribution of the radiocesium survey of edible wild plants (12 species) during 2012–2015 (Table C).
(XLSX)

## References

[1] International Atomic Energy Agency. The Fukushima Daiichi accident In: Technical Volume 1. Description and Context of the Accident. Vienna: International Atomic Energy Agency; 2015 pp. 1–225.

[2] United Nations Scientific Committee on the Effects of Atomic Radiation. Report to the general assembly, scientific annex A: levels and effects of radiation exposure due to the nuclear accident after the 2011 great East-Japan earthquake and tsunami, Volume I; 2013.

[3] Komatsu M, Kaneko S, Ohashi S, Kuroda K, Sano T, Ikeda S, et al Characteristics of initial deposition and behavior of radiocesium in forest ecosystems of different locations and species affected by the Fukushima Daiichi Nuclear Power Plant accident. J Environ Radioact. 2016; 161:2–10. doi: 10.1016/j.jenvrad.2015.09.016 26432062 10.1016/j.jenvrad.2015.09.016

[4] Teramage MT, Onda Y, Kato H and Gomi T. The role of litterfall in transferring Fukushima-derived radiocesium to a coniferous forest floor. Sci Total Environ. 2014; 490:435–9. doi: 10.1016/j.scitotenv.2014.05.034 24875259 10.1016/j.scitotenv.2014.05.034

[5] International Atomic Energy Agency. The Follow-up IAEA International Mission on Remediation of Large Contaminated Areas Off-Site the Fukushima Daiichi Nuclear Power Plant. Vienna: International Atomic Energy Agency; 2013.

[6] Fukushima Prefectural Government. Fukushima Revitalization Station. Environmental Restoration; Radiation levels in the prefecture. Available from: http://www.pref.fukushima.lg.jp/site/portal-english/en02-01.html. (Accessed 2017 July 26)(

[7] Nuclear Regulation Authority of Japan. Monitoring Information of Environmental Radioactivity Level. Available from: http://radioactivity.nsr.go.jp/en/. (Accessed 2017 July 26)

[8] Kawauchi Village Local Government. From the village head. Available from: http://translate.google.co.jp/translate?hl=ja&sl=ja&tl=en&u=http%3A%2F%2Fwww.kawauchimura.jp%2F. (Accessed 2017 July 26)

[9] Reconstruction Promotion Committee of Japan. Reconstruction Promotion Committee FY2012 Interim Report. Available from: http://www.reconstruction.go.jp/english/topics/20121228_FAINAL_CHUKAN.pdf. (Accessed 2017 July 26)

[10] Consumer Affairs Agency, Government of Japan. White Paper on Consumer Affairs. Available from: http://www.caa.go.jp/en/publications/annual_reports/2016/pdf/en_summary.pdf. (Accessed 2017 July 26)

[11] Ministry of Agriculture, Foresty and Fisheres of Japan. The Great East Japan Earthquake: Request for shipment restraint and other measures. Available from: http://www.maff.go.jp/e/policies/food_safety/emer/shipment.html. (Accessed 2017 July 26)

[12] Harada KH, Niisoe T, Imanaka M, Takahashi T, Amako K, Fujii Y, et al Radiation dose rates now and in the future for residents neighboring restricted areas of the Fukushima Daiichi Nuclear Power Plant. Proc Natl Acad Sci U S A. 2014; 111 (10): E914–23. doi: 10.1073/pnas.1315684111 24567380 10.1073/pnas.1315684111 PMC3956155

[13] Orita M, Hayashida N, Nukui H, Fukuda N, Kudo T, Matsuda N, et al Internal radiation exposure dose in Iwaki city, Fukushima prefecture after the accident at Fukushima Dai-ichi Nuclear Power Plant. PLoS One. 2014; 9 (12): e114407 doi: 10.1371/journal.pone.0114407 25478794 10.1371/journal.pone.0114407 PMC4257669

[14] Tsubokura M, Kato S, Nihei M, Sakuma Y, Furutani T, Uehara K, et al Limited internal radiation exposure associated with resettlements to a radiation-contaminated homeland after the Fukushima Daiichi nuclear disaster. PLoS One. 2013; 8 (12): e81909 doi: 10.1371/journal.pone.0081909 24312602 10.1371/journal.pone.0081909 PMC3846705

[15] Tsubokura M, Kato S, Nomura S, Morita T, Sugimoto A, Gilmour S, et al Absence of internal radiation contamination by radioactive cesium among children affected by the Fukushima Daiichi nuclear power plant disaster. Health Phys. 2015; 108 (1): 39–43. doi: 10.1097/HP.0000000000000167 25437518 10.1097/HP.0000000000000167

[16] Kim E, Kurihara O, Kunishima N, Momose T, Ishikawa T and Akashi M. Internal thyroid doses to Fukushima residents—estimation and issues remaining. J Radiat Res. 2016; 57 (Suppl 1): i118–i126. doi: 10.1093/jrr/rrw061 27538842 10.1093/jrr/rrw061 PMC4990119

[17] World Health Organization. Health risk assessment from the nuclear accident after the 2011 Great East Japan Earthquake and Tsunami based on a preliminary dose estimation; 2013.

[18] Ikeda Y. Cooking edible wild plants and herbs by Akira Ueno and Yoko Terada. Kurashinosekkei 157, Chuokoron-sha, Tokyo; 1984. pp. 168 (in Japanese).

[19] Kiyono Y and Akama A. Radioactive cesium contamination of edible wild plants after the accident at the Fukushima Daiichi Nuclear Power Plant. Jpn J For Environ. 2013; 55 (2): 113–118.

[20] The Japan Times. LIFE. Available from: http://www.japantimes.co.jp/life/2015/09/05/lifestyle/feast-forest-foraging-edible-plants-japan/#.WSTcM7puKUk. (Accessed 2017 July 26)

[21] Orita M, Nakashima K, Taira Y, Fukuda T, Fukushima Y, Kudo T, et al Radiocesium concentrations in wild mushrooms after the accident at the Fukushima Daiichi Nuclear Power Station: Follow-up study in Kawauchi village. Sci Rep. 2017; 7 (1): 6744 doi: 10.1038/s41598-017-05963-0 28751728 10.1038/s41598-017-05963-0 PMC5532244

[22] Nakashima K, Orita M, Fukuda N, Taira Y, Hayashida N, Matsuda N, et al Radiocesium concentrations in wild mushrooms collected in Kawauchi Village after the accident at the Fukushima Daiichi Nuclear Power Plant. PeerJ. 2015; 3: e1427 doi: 10.7717/peerj.1427 26623189 10.7717/peerj.1427 PMC4662580

[23] Orita M, Hayashida N, Taira Y, Fukushima Y, Ide J, Endo Y, et al Measurement of individual doses of radiation by personal dosimeter is important for the return of residents from evacuation order areas after nuclear disaster. PLoS One. 2015; 10 (3): e0121990 doi: 10.1371/journal.pone.0121990 25806523 10.1371/journal.pone.0121990 PMC4373698

[24] Orita M, Nakashima K, Hayashida N, Endo Y, Yamashita S and Takamura N. Concentrations of Radiocesium in Local Foods Collected in Kawauchi Village after the Accident at the Fukushima Dai-ichi Nuclear Power Station. Sci Rep. 2016; 6: 28470 doi: 10.1038/srep28470 27334847 10.1038/srep28470 PMC4917854

[25] Taira Y, Hayashida N, Orita M, Yamaguchi H, Ide J, Endo Y, et al Evaluation of environmental contamination and estimated exposure doses after residents return home in Kawauchi Village, Fukushima Prefecture. Environ Sci Technol. 2014; 48 (8): 4556–63. doi: 10.1021/es404534y 24641663 10.1021/es404534y

[26] Ministry of Education, Culture, Sports, Science, and Technology of Japan. Radioactivity measurement series. Available from: http://www.jcac.or.jp/site/library/series.html. (Accessed 2017 July 26)

[27] Information on the Great East Japan Earthquake. Food. Available from: http://www.mhlw.go.jp/english/topics/2011eq/dl/food-130926_1.pdf. (Accessed 2017 July 26)

[28] International Commission on Radiological Protection. Age-dependent Doses to the Members of the Public from Intake of Radionuclides—Part 5 Compilation of Ingestion and Inhalation Coefficients. ICRP Publication 72, Annals of the ICRP; 1995.

[29] Ministry of Health, Labour, and Welfare of Japan. The second term of National Health Promotion Movement in the twenty first century, National Health and Nutrition Survey; Nutritional Intake Status Survey; Intake by Food Groups; Vegetables. Available from: http://www.mhlw.go.jp/seisakunitsuite/bunya/kenkou_iryou/kenkou/kenkounippon21/en/eiyouchousa/kekka_syokuhin_chousa_koumoku.html. (Accessed 2017 July 26)

[30] World Health Organization. Global Environment Monitoring System (GEMS/food); 1976. Available from: http://www.who.int/foodsafety/areas_work/chemical-risks/gems-food/en/. (Accessed 2017 July 26)

[31] Ministry of Health, Labour, and Welfare of Japan. Information on the Great East Japan Earthquake. Food. (Available from: http://www.mhlw.go.jp/english/topics/2011eq/dl/food-130926_1.pdf. (Accessed 2017 July 26)

[32] Ministry of Health, Labour, and Welfare of Japan. New Standard limits for Radionuclides in Foods (provisional translation); Department of Food Safety, Pharmaceutical & Food Safety Bureau, the Ministry of Health Labour and Welfare. Available from: http://www.mhlw.go.jp/english/topics/2011eq/dl/new_standard.pdf. (Accessed 2017 July 26)

[33] Ministry of Agriculture, Foresty and Fisheres of Japan. The Great East Japan Earthquake. (in Japanese) Available from: http://www.rinya.maff.go.jp/j/tokuyou/kinoko/sansai.html. (Accessed 2017 July 26)

[34] International Commission on Radiological Protection. Age-dependent Doses to Members of the Public from Intake of Radionuclides—Part 2 Ingestion Dose Coefficients. ICRP Publication 67, Annals of the ICRP, vol. 22, Nos. 3–4; 1992. 7978694

[35] Taira Y, Hayashida N, Yamaguchi H, Yamashita S, Endo Y and Takamura N. Evaluation of environmental contamination and estimated radiation doses for the return to residents' homes in Kawauchi Village, Fukushima Prefecture. PLoS ONE. 2012; 7 (9): e45816 doi: 10.1371/journal.pone.0045816 23049869 10.1371/journal.pone.0045816 PMC3458921

[36] Taira Y, Hayashida N, Yamashita S, Kudo T, Matsuda N, Takahashi J, et al Environmental contamination and external radiation dose rates from radionuclides released from the Fukushima Nuclear Power Plant. Radiat Prot Dosim. 2012; 151 (3): 537–545. doi: 10.1093/rpd/ncs040 22504310 10.1093/rpd/ncs040

[37] Portal Site of Official Statistics of Japan (e-Stat). Search result top page; System of Social and Demographic Statistics; Social Indicators by Prefecture 2017; Social Indicators by Prefecture; Natural Environment. (Available from: http://www.e-stat.go.jp/SG1/estat/GL32020101.do?method=extendTclass&refTarget=toukeihyo&listFormat=hierarchy&statCode=00200502&tstatCode=000001095536&tclass1=&tclass2=&tclass3=&tclass4=&tclass5=. (Accessed 2017 July 26)

[38] Loffredo N, Onda Y, Hurtevent P and Coppin F. Equation to predict the ^137^Cs leaching dynamic from evergreen canopies after a radio-cesium deposit. J Environ Radioact. 2015; 147: 100–107. 10.1016/j. doi: 10.1016/j.jenvrad.2015.05.018 26057986 10.1016/j.jenvrad.2015.05.018

[39] Ohashi S, Okada N, Tanaka A, Nakai W and Takano S. Radial and vertical distributions of radiocesium in tree stems of Pinus densiflora and Quercus serrata 1.5 y after the Fukushima nuclear disaster. J Environ Radioact. 2014; 134: 54–60. doi: 10.1016/j.jenvrad.2014.03.001 24661964 10.1016/j.jenvrad.2014.03.001

[40] Yoschenko V, Takase T, Konoplev A, Nanba K, Onda Y, Kivva S, et al Radiocesium distribution and fluxes in the typical Cryptomeria japonica forest at the late stage after the accident at Fukushima Dai-Ichi Nuclear Power Plant. J Environ Radioact. 2017; 166 (1): 45–55. 10.1016/j. 26948679 10.1016/j.jenvrad.2016.02.017

[41] National Council on Radiation Protection and Measurements. Report No. 161 I—Management of Persons Contaminated With Radionuclides: Handbook; 2008. Available from: https://www.ncrppublications.org/Reports/161_I. (Accessed 2017 July 26)

[42] Taira Y, Hayashida N, Brahmanandhan GM, Nagayama Y, Yamashita S, Takahashi J, et al Current concentration of artificial radionuclides and estimated radiation doses from ^137^Cs around the Chernobyl Nuclear Power Plant, the Semipalatinsk Nuclear Testing Site, and in Nagasaki. J Radiat Res. 2011; 52 (1): 88–95. 21187665 10.1269/jrr.10104

[43] Hosokawa Y, Nomura K, Tsushima E, Kudo K, Noto Y and Nishizawa Y. Whole-Body Counter (WBC) and food radiocesium contamination surveys in Namie, Fukushima Prefecture. PLoS One. 2017; 12 (3): e0174549 doi: 10.1371/journal.pone.0174549 28334042 10.1371/journal.pone.0174549 PMC5363944

[44] Orita M, Fukushima Y, Yamashita S and Takamura N. The Need for Forest Decontamination: For the Recovery of Fukushima. Radiat Prot Dosimetry. 2016; 175 (2): 295–296. doi: 10.1093/rpd/ncw301 27886984 10.1093/rpd/ncw301

[45] Yoshida K, Shinkawa T, Urata H, Nakashima K, Orita M, Yasui K, et al Psychological distress of residents in Kawauchi village, Fukushima Prefecture after the accident at Fukushima Daiichi Nuclear Power Station: the Fukushima Health Management Survey. PeerJ. 2016; 4: e2353 doi: 10.7717/peerj.2353 27635326 10.7717/peerj.2353 PMC5012316

